# A Novel Ultrasound-Assisted Extraction Method for the Analysis of Anthocyanins in Potatoes (*Solanum tuberosum* L.)

**DOI:** 10.3390/antiox10091375

**Published:** 2021-08-28

**Authors:** Ceferino Carrera, María José Aliaño-González, Monika Valaityte, Marta Ferreiro-González, Gerardo F. Barbero, Miguel Palma

**Affiliations:** 1 Department of Analytical Chemistry, Faculty of Sciences, Agrifood Campus of International Excellence (ceiA3), IVAGRO, University of Cadiz, 11510 Puerto Real, Spain; ceferino.carrera@uca.es (C.C.); mariajose.alianogonzalez@alum.uca.es (M.J.A.-G.); marta.ferreiro@uca.es (M.F.-G.); miguel.palma@uca.es (M.P.); 2 Department of Biology, Faculty of Marine and Environmental Sciences, University of Cadiz, 11510 Puerto Real, Spain; mvalaityte@yahoo.com

**Keywords:** anthocyanins, Box-Behnken design, purple potato, red potato, *Solanum tuberosum*, ultrasound-assisted extraction, optimization, UHPLC, antioxidant activity, DPPH

## Abstract

Purple potato is one of the least known and consumed potato varieties. It is as rich in nutrients, amino acids and starches as the rest of the potato varieties, but it also exhibits a high content of anthocyanins, which confer it with some attractive health-related properties, such as antioxidant, pain-relieving, anti-inflammatory and other promising properties regarding the treatment of certain diseases. A novel methodology based on ultrasound-assisted extraction has been optimized to achieve greater yields of anthocyanins. Optimal extraction values have been established at 70 °C using 20 mL of a 60% MeOH:H_2_O solution, with a pH of 2.90 and a 0.5 s^−1^ cycle length at 70% of the maximum amplitude for 15 min. The repeatability and intermediate precision of the extraction method have been proven by its relative standard deviation (RSD) below 5%. The method has been tested on Vitelotte, Double Fun, Highland and Violet Queen potatoes and has demonstrated its suitability for the extraction and quantification of the anthocyanins found in these potato varieties, which exhibit notable content differences. Finally, the antioxidant capacity of these potato varieties has been determined by means of 2,2-diphenyl-1-picrylhydrazyl (DDPH) radical scavenging and the values obtained were similar to those previously reported in the literature.

## 1. Introduction

Potato (*Solanum tuberosum* L.) is one of the most extensively consumed food products around the world, as it is an important source of nutrients and calories with a reduced content of undesirable compounds, such as fat or salt, and a high content of essential amino acids and starches [1,2,3,4].

There are a large number of potato varieties, each of them with specific chemical and physical profiles, the white, cream, or yellow flesh varieties being the most widely consumed [5,6]. However, other varieties such as red or purple flesh potatoes are not so well known, as consumers are not so familiar with their color, and they tend to be included in some gourmet dishes [7].

Different research studies have been carried out over the last few years reporting a number of important properties—antioxidant, anti-inflammatory or prebiotic—associated with purple flesh potatoes, which belong to the same species [8,9,10]. They also favor mitochondrial functions with significant effects against obesity, diabetes or other metabolic disorders, and cancer inhibitory effects have also been reported [11,12,13,14,15,16,17]. A deeper study on the composition of purple potatoes discovered a large concentration of dietary fiber, minerals, vitamins, flavonoids, anthocyanins and phenolic compounds that are associated with several of the above-mentioned properties exhibited by potatoes [18,19].

Anthocyanins are natural pigments from the phenolic compound family [20,21] that confer a red, purple or blue color to many vegetables or fruits such as blueberries, açai, grapes, onions, sloes or even purple potatoes [22,23,24,25,26,27,28]. Anthocyanins are chemical compounds of high interest because they can directly scavenge reactive oxygen species (ROS), modulate the synthesis or activity of some antioxidant enzymes or even have an influence on mitochondrial respiration to prevent ROS generation or a decisive effect on the cellular antioxidant system [29,30,31]. Additionally, anthocyanins have exhibited significant activities in the reduction of inflammation markers from illnesses such as cancer, cardiovascular diseases, obesity or neurodegenerative diseases [32,33,34,35], which represents an important advance in preventing and treating these disorders. Furthermore, anthocyanins have proved an important relationship with pain mitigation [36]. Therefore, counting with effective methodologies that allow the extraction of anthocyanins from purple potatoes efficiently would be of utmost importance for its critical application in medicine, cosmetics and pharmaceutical industries or even in the food industry as a natural dye [37,38].

Different techniques have been employed for the extraction and analysis of anthocyanins from purple potatoes, acetylated derivatives of malvidin, petunidin, peonidin and delphinidin being the main anthocyanins that have been identified up to date [39,40,41].

Ultrasound-assisted extraction (UAE) has been demonstrated to be an efficient method for extracting bioactive compounds from different natural sources, which includes blackberry, lavender, raspberry, pepper, walnut, onion or even purple potato [28,42,43,44,45,46,47]. This is due to its many advantages, such as shorter extraction times, less solvent consumption and lower production costs with higher yields, reaching even twice the extraction yield, in comparison to conventional extraction techniques [48,49,50,51]. In addition, it is quite easy to apply, cost-effective and does not require complex maintenance operations [52]. This extraction technique is based on the phenomenon of cavitation, a mechanical effect caused by ultrasounds, that instigate mass transfer and compound extraction [53,54] at low temperature and with short extraction times. Hence, the compounds of interest, such as anthocyanins, are not degraded because of the extraction procedure, and larger yields are obtained [55].

An efficient method for the extraction of anthocyanins from purple potato depends on a number of factors that exert a varying degree of influence on the process. These factors include solvent properties, extraction temperature and time, among others. In order to obtain extracts enriched with anthocyanins from purple potato that can be used for the previously mentioned purposes, it is important to know the optimal conditions for the extraction that guarantee the highest performance.

In the present research, a Box-Behnken design (BBD) combined with response surface methodology (RSM) was applied to investigate the extraction of anthocyanins from purple potatoes. BBD-RSM is a mathematical and statistical method that evaluates how a number of selected variables affect the efficiency of the anthocyanin extraction method so that it can be optimized. In addition, ultra-high-performance liquid chromatography (UHPLC) coupled to ultraviolet-visible spectroscopy was selected as the method to quantify anthocyanin extractions from purple potatoes.

In conclusion, this research intends to establish the optimal conditions to achieve the maximum possible extraction of anthocyanins from purple potato samples using UAE, a technique that presents multiple advantages in comparison to other conventional methods. The objective of the authors is to develop a novel methodology that can be applied to verify the quality not only of the raw material but also of the intermediate and final products that can be obtained from purple potatoes. The final method should be suitable for industries, as well as for their analysis by commercial laboratories that intend to verify the composition and quality of any of the main or derived products that can be obtained from purple potatoes.

## 2. Materials and Methods

### 2.1. Biological Material

The flesh from purple potatoes of the Vitelotte variety acquired from a market in Cadiz (province of Andalusia, Spain) was used to design and optimize the extraction and analysis methodologies. Seed potatoes of the Vitelotte, Double Fun, Violet Queen (purple potatoes) and Highland (red potato) varieties were purchased from Fitoagricola.net (Castellón, Spain) and grown at the same plot (Torrecera, Spain; N: 36°36′39.9′′; W: 5°56′22.6′′) from January until May 2021.

In all the cases, the skin and bulbs were removed, and the flesh was cut into small pieces with the aim of quantifying the anthocyanins content in this part of the potatoes. After that, the samples were lyophilized using an LYOALFA 10/15 freeze dryer (Telstar Technologies, S.L., Terrassa, Barcelona, Spain) until a constant weight was reached. Finally, the samples were crushed by means of a ZM200 knife mill (Retsch GmbH, Haan, Germany) and stored in a freezer at −20 °C until further analysis.

### 2.2. Chemical and Solvents

The solvents chosen for the extraction were methanol of HPLC purity (Fisher Scientific, Loughborough, UK) and Milli-Q water, acquired from a Milli-Q water purification system (Millipore, Bedford, MA, USA). The extraction solvents were prepared in liquid-liquid mixtures at different percentages, and the pH values were adjusted using HCl (1 M) and NaOH solution (0.5 M) (Panreac, Barcelona, Spain).

For the UHPLC analysis, methanol of HPLC grade (Fisher Scientific, Loughborough, UK) and formic acid of HPLC grade (Panreac, Barcelona, Spain) were used for chromatographic separation.

A commercial standard, namely cyanidin chloride (≥95% purity, Sigma-Aldrich Chemical Co., St. Louis, MO, USA), was used for the quantification of the anthocyanins in the extracts.

Finally, the antioxidant activity of the yields was evaluated by 2,2-diphenyl-1-picrylhydrazyl (DPPH) radical scavenging and Tris base, both supplied by Sigma-Aldrich (St. Louis, MO, USA). The standard used for control was 6-hydroxy-2,5,7,8-tetramethylchroman-2-carboxylic acid (Trolox) from Sigma-Aldrich (Steinheim, Germany).

### 2.3. Ultrasound-Assisted Extraction

#### 2.3.1. Ultrasound-Assisted Extraction Equipment

For the ultrasound-assisted extraction, a UP200S sonifier (200 W, 24 kHz) (Dr. Hielscher, GmbH, Berlin, Germany) was employed. This equipment is fitted with an amplitude controller, a cycle controller and a water bath equipped with a temperature controller (FRIGITERM-10, J.P. Selecta, S.A., Barcelona, Spain).

#### 2.3.2. Ultrasound-Assisted Extraction Optimization

As previously mentioned, BBD-RSM was the method chosen for the optimization of the UAE extraction. In this type of design, three levels per factor are evaluated: (−1) a lower level, (0) an intermediate level and (1) a higher level. In addition, it does not present axial points so that it has a more spherical arrangement of the design points than other statistical designs. Consequently, not only is a lower total number of experiments required, but also any experiments to be run under extreme conditions and that might cause the degradation of the anthocyanins or that may represent an excessive financial expense are excluded [56].

Four independent factors were selected to optimize the performance of the equipment: (i) composition of the extraction solvents (%MeOH in Milli-Q water; 0, 25 and 50%), (ii) extraction temperature (10, 40 and 70 °C), (iii) pH of the solvent (2, 4.5 and 7) and (iv) sample-to-solvent ratio (1:100, 1:150 and 1:200 g/mL). The cycle was set up at 0.5 s^−1^ and the amplitude at 70% of the ultrasound equipment’s maximum amplitude (200 W). These values and ranges were assigned based on the research team’s previous experience on similar matrices, as well as on the relevant bibliography [57,58,59,60]. The resulting design comprised a total of 27 extractions, including 3 repetitions at the center point, and all the experiments were performed at random.

Regarding the response variable, the area of the identified anthocyanins was measured and normalized according to the amount of sample weighted. The sum of these normalized areas was taken as the response variable. A summary of the experimental conditions for the BBD-RSM (Table 1).

The extracts obtained from each experiment were centrifuged twice for 5 min at 5985× *g*, filtered using a 0.20 μm nylon syringe filter (Membrane Solutions, Dallas, TX, USA) and analyzed under the conditions below.

### 2.4. Identification of Anthocyanins by UHPLC-PDA-QToF-MS

An ultra–high-performance liquid chromatography equipment coupled to a photodiode array detector and to a quadrupole time-of-flight mass spectrometry (UHPLC-PDA-QToF-MS) model Xevo G2 (Waters Corp., Milford, MA, USA) was employed for the identification of the anthocyanins from the purple and red potato samples. This system contains a 100 × 2.1 mm reverse-phase C18 analytical column (Acquity UPLC BEH C18, Waters) with a particle size of 1.7 µm. Phase A contained water and formic acid at 2%, and phase B was pure methanol. The flow rate was 0.4 mL/min. The injection gradient employed was 5% B at 0 min; 20% B at 3.30 min; 30% B at 3.86 min; 40% B at 5.05 min; 55% B at 5.35 min; 60% B at 5.64 min; 95% B at 5.94 min; 95% B at 7.50 min. In summary, a total time of 12 min was required for each analysis, including 4 min for re-equilibration. The electrospray was applied in positive ionization mode. The desolvation gas temperature was 500 °C with a flow of 700 L/h, and the capillary cone was set at 700 V. On the other hand, the cone gas flow was 10 L/h, the temperature of the source was 150 °C and the cone voltage was 20 V. Finally, the trap collision energy was 4 eV. Full-scan mode in a 100–1200 *m*/*z* range was employed for the identification of the anthocyanins

The major anthocyanins identified in the purple potato extracts were [M^+^]: 2. Petunidin 3-*p*-coumaroylrutinoside-5-glucoside (*m*/*z* 933.2390); 4. Peonidin 3-*p*-coumaroylrutinoside-5-glucoside (*m*/*z* 917.2419); 5. Malvidin 3-*p*-coumaroylrutinoside-5-glucoside (*m*/*z* 947.2563); 7. Malvidin 3-feruoylrutinoside-5-glucoside (*m*/*z* 977.2499). The major anthocyanins identified in the red potato extracts were [m^+^]: 1. Pelargonidin 3-*p*-coumaroylrutinoside-5-glucoside (*m*/*z* 887.2379); 3. Pelargonidin 3-*p*-coumaroylrutinoside-5-glucoside (*m*/*z* 887.2385); 6. Pelargonidin derivative (*m*/*z* 887.2387).

The identified anthocyanins are in agreement with those described by Gutierrez-Quequezana et al. [42] and by Kita et al. [61]. The mass chromatograms of the identified anthocyanins are included in the supplementary material (Figures S1 and S2).

### 2.5. Separations and Quantification of the Anthocyanins by UHPLC–UV–Vis

Once anthocyanins were identified, their separation and quantification in the purple and red potato samples was conducted by means of an Elite UHPLC LaChrom System (Hitachi, Tokyo, Japan). This system features an L–2200U autosampler, an L2300 column oven, which was set up at 50 °C, two L–2160U pumps and a UV–Vis detector L–2420U. The last one was set at 520 nm for identification purposes. A reverse–phase C18 (Phenomenex, Kinetex, CoreShell Technology, Torrance, CA, USA) 2.1 × 50 mm and 2.6 µm particle size column was employed. As mentioned above, phase A was water with 5% of formic acid, and phase B was pure methanol. In order to avoid impurities and possible bubbles, both solvents were filtered using a 0.22 µm filter (RephiLe Bioscience, Ltd., Shanghai, China) and degassed using an ultrasonic bath (Elma S300, Elmasonic, Singen, Germany). The flow rate was 0.7 mL/min.

A 0.22 µm nylon syringe filter (Membrane Solutions, Dallas, TX, USA) was used for filtering the extracts before their analysis. The injection volume was set at 15 μL, and the time and % of solvent B for the UHPLC separation were the following: 0.00 min, 2%; 1.50 min, 2%; 3.30 min, 15%; 4.80 min, 15%; 5.40 min, 35%; 6 min, 100%.

This method was chosen because it allows the separation of the seven major anthocyanins identified in the samples in less than 7 min. Short-time analysis represents an important advantage, particularly for quality control laboratories, where hundreds of analyses are to be completed on a daily basis.

Cyanidin chloride was selected as a reference standard; the calibration curve obtained (y = 300,568.88x − 28,462.43) exhibited a coefficient of regression R^2^ = 0.9999. Furthermore, the limits of detection (LOD) and quantification (LOQ) were 0.198 mg L^−1^ and 0.662 mg L^−1^, respectively. The normal distribution of residuals was tested by Shapiro-Wilk test, and a W value of 0.8514 (very close to 1) was obtained, as well as a *p*-value of 0.803 (above 0.05), which confirms hypothesis H_o_. Because different anthocyanins show similar absorbance values and considering the individual molecular weights, this calibration curve was used to quantify the different anthocyanin contents in the potato extract samples. All the analyses were performed in duplicate.

### 2.6. DPPH Analysis

The antioxidant activity of the anthocyanins found in purple and red potato samples was evaluated using DPPH assays based on the procedure designed by Brand-Williams et al. [62] with the modifications implemented by Miliauskas et al. [63]. The α-diphenyl-*β*-picrylhydrazyl (DPPH; C_18_H_12_N_5_O_6_) molecule is characterized by the delocalization of the spare electron in the molecule, which is the reason why these molecules do not dimerize like most other free radicals do [64]. This specific characteristic makes DPPH a stable free radical that exhibits a violet color in the solution due to a strong absorption band at about 515 nm. When DPPH is in the presence of a substance that can donate a hydrogen atom (an antioxidant substance), the odd electron from the nitrogen atom in DPPH is reduced. This causes a change in the color of the solution from violet into pale yellow that can be measured at 515 nm and that can be used to determine the antioxidant activity of any compound that is mixed with it [59].

For this research, a 6 × 10^−5^ M DPPH solution in methanol was prepared. A 2 mL volume of this solution was mixed with 100 µL of purple or red potato extract and with 900 µL of Tris-HCl buffer (0.1 M at pH 7.4). The mixture was kept at room temperature in the absence of light for 40 min. After that, the absorbance of the solution was measured at 515 nm.

Trolox was selected as the standard, and a six-point linear regression model was calculated from 0 until 1.4 mM in triplicate. The regression equation obtained (y = 88.94x + 0.75) exhibited a determination coefficient R^2^ = 0.9959. The antioxidant activity was expressed as mg of Trolox equivalents (TE) per gram of dry weight potato (mg TE g^−1^ DW).

### 2.7. Optimization Study

After completing the 27 extractions for the BBD-RSM and evaluating the extraction variables of the total anthocyanins extractions, a second-order polynomial equation, where all the variables were considered, was applied.

The polynomial equation is the following:

Y = *β*_0_ + *β*_1_X_1_ + *β*_2_X_2_ + *β*_3_X_3_ + *β*_4_X_4_ + *β*_12_X_1_X_2_ + *β*_13_X_1_X_3_ + *β*_14_X_1_X_4_ + *β*_23_X_2_X_3_ + *β*_24_X_2_X_4_ + *β*_34_X_3_X_4_ + *β*_11_X_1_^2^ + *β*_22_X_2_^2^ + *β*_33_X_3_^2^ + *β*_44_X_4_^2^ (1)

where Y is the aforesaid response, and *β*_0_ corresponds to the ordinate, whereas X_1_ (% MeOH in the solvent), X_2_ (extraction temperature), X_3_ (pH of the solvent) and X_4_ (sample-to-solvent ratio) are independent variables. Finally, *β*_i_ are the linear coefficients, *β*_ij_ are the cross-product coefficients and *β*_ii_ are the quadratic coefficients.

The effect of each factor and of their interactions on the response variable, the second-order mathematical model, the surface graphs, the optimal levels of the significant variables and the variance analysis were calculated by means of Statgraphic Centurion software (version XVII) (Statgraphics Technologies, Inc., The Plains, VA, USA) and Minitab software (version X) (Minitab LLC, State College, PA, USA). After that, repeatability and intermediate precision tests under the established optimum conditions were conducted. For the repeatability analysis, eight extractions were performed on the same day, and for the intermediate precision, eight extractions were conducted on three days in a row (a total of 24 extractions). The coefficient of variation was the statistical parameter used as the reference to determine the repeatability and intermediate precision levels of the optimized method.

## 3. Results and Discussion

### 3.1. Optimization of the Method

Four variables were selected to optimize the UAE methodology in order to maximize anthocyanins recoveries from purple potato samples. The BBD-RSM was employed, and a total of 27 extracts were obtained under different conditions and analyzed by UHPLC-UV-vis. The measured and predicted values were correlated (Table 1), and an average 7.3% difference was obtained, ranging between 0.18% and 17.7%.

The model obtained showed an R-squared statistic of 0.89, and the *p*-value for Durbin-Watson in the ANOVA (0.257) was above 0.05, which means that there are no significant differences between the predicted and the observed values.

The *t*-test using Minitab software with a 95% confidence level was applied to obtain the *p*-values for each of the optimized variables. Consequently, the variables presenting values of *p* < 0.05 were selected as influential. These data can be seen in Table 2.

As can be observed in Table 2, the percentage of methanol (*p* = 0.00), the solvent pH (*p* = 0.01) and the extraction temperature (*p* = 0.00) exhibited a clear influence on the normalized area of the anthocyanins. The ratio was the only independent variable that did not significantly influence anthocyanin extraction from purple potato samples. On the other hand, the quadratic interaction of the solvent pH (*p* = 0.02) was the only interaction with a significant influence as well. The rest of the interactions between the optimized variables did not have a significant influence on the extractions.

**Figure 1:**
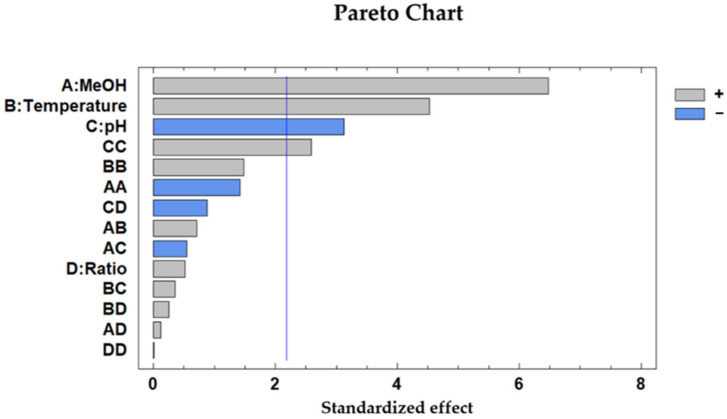
Pareto chart corresponding to the BBD-RSM analyses of the anthocyanins in the purple potato sample extracts. A: %MeOH in the solvent; B: extraction temperature; C: extraction solvent pH; D: sample-to-solvent ratio.

In order to determine the influence from each variable, a Pareto chart was elaborated (Figure 1), and a positive correlation with the percentage of solvent (methanol), the temperature and the quadratic interaction of the solvent pH was observed, while solvent pH had a negative effect on the recovery of the anthocyanins.

The second-order polynomial equation to calculate the total extraction of anthocyanins under the established optimal conditions according to the coefficients resulting from the BBD–RSM analyses is:

Y = 114,049.0 + 106,440.0X_1_ + 74,471.3X_2_ − 51,482.7X_3_ + 8528.2X_4_ + 20,227.2X_1_X_2_ − 15,495.5X_1_X_3_ + 3523.3X_1_X_4_ + 10,062.5X_2_X_3_ + 7089.9X_2_X_4_ − 25,124.8X_3_X_4_ − 35,089.0X_1_^2^ + 36,446.9X_2_^2^ + 63,815.8X_3_^2^ + 197.1X_4_^2^ (2)

**Figure 2:**
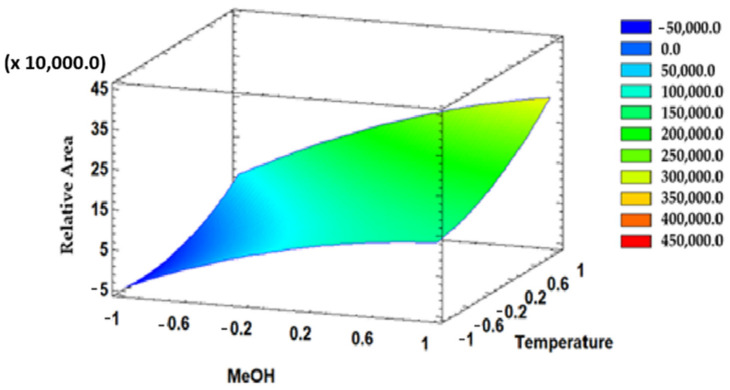
Surface response diagram representing the effect of temperature and percentage of methanol in the solvent with respect to the relative area of the anthocyanins extracted.

Extraction temperature and solvent pH were established as influential variables, and the interaction between them was evaluated by means of a surface response graph (Figure 2). The dark blue areas represent the lowest relative areas, whereas the highest relative area appears in light green color and corresponds to the conditions required for the largest possible anthocyanins recovery. In this case, the area representing the maximum anthocyanins recovery was obtained when the solvent pH and the temperature were close to the highest values in the range.

The optimal conditions for the maximum recovery of anthocyanins from purple potato were as follows: 0.5 g of sample extracted adding 20 mL of solvent at 50% methanol in water at pH 2.90 and at 70 °C. These conditions were in agreement with those obtained by other authors when extracting anthocyanins from other matrices [28,46]. The optimal temperature was at the highest end of the range studied (70 °C). Given that higher temperature levels could make the solvent boil and affect the final recovery, the authors decided not to run any tests beyond the established range.

The percentage of methanol in the solvent was at the highest value within the range as well. On the other hand, because some authors have observed that greater extraction of anthocyanins is achieved when methanol percentages are above 50%, especially from plant matrices [30,65,66], higher methanol concentrations were also tested (50–100%).

These extractions were carried out under the previously established optimal conditions (0.5 g of sample, 70 °C, 20 mL of solvent with a pH of 2.90), while the percentage of methanol was being modified. Each extraction was carried out in triplicate, and the total response (total anthocyanins) was registered as previously explained.

**Figure 3:**
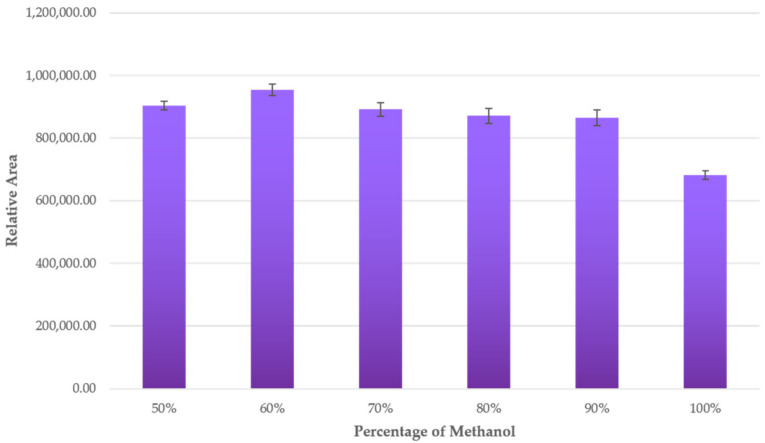
Anthocyanins’ relative area according to different methanol percentages in the extraction solvent (*n* = 3).

The relative area of the anthocyanins when different percentages of methanol were used is shown in Figure 3. Standard deviations of data were included as error bars. Thus, it was observed that the relative area reached its maximum level when the percentage of methanol was 60%, with significant differences to the rest of the percentages evaluated. At values above 60%, the relative area went down, with significant differences when methanol concentration was between 70% and 100%. Finally, the minimum relative area was registered at the maximum percentage of methanol concentration (100%). For this reason, it was determined that the optimum percentage of methanol in the solvent was 60%.

### 3.2. Optimal Extraction Time of the Method

**Figure 4:**
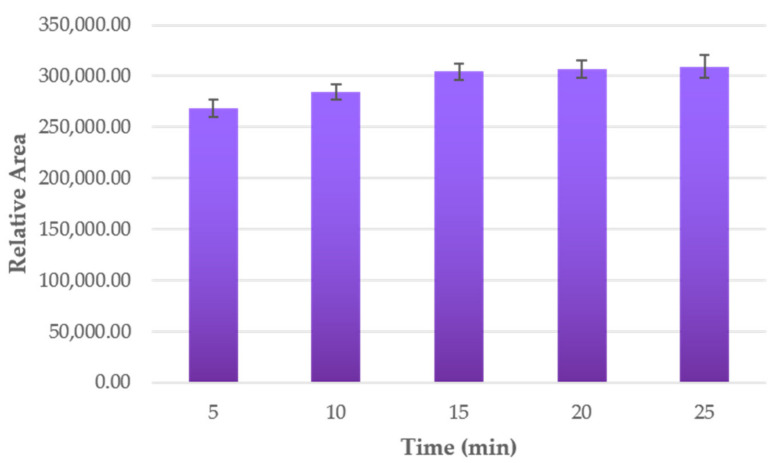
Anthocyanins’ relative area according to the different extraction times (*n* = 3).

Once the optimal extraction conditions were established, the optimal extraction time was to be determined. For that purpose, 0.5 g of purple potato sample was extracted at 70 °C using 20 mL of solvent formed by 60% methanol in water with 2.90 pH and a 0.5 s^−1^ cycle at 70% of the ultrasound maximum amplitude. For these extraction runs, different times were studied from 5 up to 25 min. All the extractions were run in triplicate, and the relative area was the response variable used to determine optimal time. Results can be found in Figure 4. Standard deviations of data were included as error bars.

A notable increment in the relative area of the anthocyanins was observed when the time was increased, with significant differences between 5, 10 and 15 min, achieving the maximum relative area at 15 min of extraction. However, no significant differences could be noticed when longer times were used. Consequently, 15 min of extraction was established as the optimum time for extracting anthocyanins from purple potato samples.

### 3.3. Repeatability and Intermediate Precision

Once the method was optimized, the following step consisted of evaluating its repeatability and intermediate precision to ensure the accuracy and precision for the detection and quantification of anthocyanins for quality control purposes of the raw materials and other products derived from purple potatoes. Thus, for the repeatability evaluation, eight extractions were carried out on the same day by applying the established optimal conditions. Likewise, for the intermediate precision assessment, eight extractions were performed on two different days up to a total of 24 extractions (*n* = 8 + 8 + 8). The average relative area, together with the residual relative standard deviation (RSD) resulting from these extractions, is presented in Table 3.

As can be observed, the RSD resulting from the repeatability and intermediate precision tests were 2.63% and 3.41%, respectively. Because both of them were below 5%, it was confirmed that the optimized method for the extraction of anthocyanins from purple potato exhibited a high precision level.

### 3.4. Re-Extraction Analysis

Once the extraction method was developed, in order to determine its effectiveness, a re-extraction study was carried out. To do so, the resulting residue was re-extracted after the double centrifugation of the extract. This residue was again subjected to an extraction process under the optimal conditions of the method. The extracts obtained were analyzed to determine the concentration of anthocyanins in this second extraction cycle. The test was carried out in triplicate. After analyzing the extracts, a concentration of 3.16% was obtained with respect to the amount of anthocyanins obtained in the first extraction cycle. This amount is less than 5%, so it is considered that one extraction cycle is sufficient for a qualitative extraction of the anthocyanins present in the potato by using the method developed by UAE.

### 3.5. Applying the Optimized Method to Different Potato Varieties

The anthocyanins contained in the four potato varieties cultivated (Vitelotte, Double Fun, Highland and Violet Queen) were extracted by applying the optimized method. For this purpose, 0.5 g of samples from each variety was subjected to the developed extraction method using 20 mL of solvent (60:40 MeOH:H_2_O, pH 2.90) at 70 °C with a 0.5 s^−1^ cycle and at 70% of the maximum amplitude for 15 min. These extractions were performed in triplicate.

UHPLC–UV–vis was used for anthocyanin quantification and the DPPH methodology for the evaluation of the antioxidant activity of the extracts. The results are displayed in Table 4. It can be observed that the purple varieties exhibit a larger anthocyanin content than the red variety (Highland). In addition, their anthocyanin profile was also different, as the purple varieties contained mainly petunidin and malvidin derivatives, while the red variety content was mostly formed of pelargonidin derivatives. On the other hand, malvidin 3-*p*-coumaroylrutinoside-5-glucoside was the main anthocyanin present in the Vitelotte and Double Fun varieties, whereas petunidin 3-*p*-coumaroylrutinoside-5-glucoside was the most abundant anthocyanin in Violet Queen’s samples (Figure S3). A one-way ANOVA was performed, and differences between data are represented in Table 4. As can be observed, significant differences were observed in petunidin 3-*p*-coumaroylrutinoside-5-glucoside between the Violet Queen variety and Vitelotte and Double Fun varieties. Significant differences were also detected in malvidin 3-*p*-coumaroylrutinoside-5-glucoside and malvidin 3-feruoylrutinoside-5-glucoside. The red variety showed the lowest anthocyanin concentration, but significant differences between the purple varieties were also detected according to the total anthocyanin content.

Regarding their antioxidant activity, it was observed that the purple flesh varieties exhibited a notably higher mg TE g^−1^ DW than the varieties with red flesh. This result agrees with those found in the literature [67,68]. In addition, the values obtained were within the same range as those reported by other authors. For example, the Vitelotte variety exhibited 28.81 mg TE g^−1^ DW, with 14.4–42.4 mg TE g^−1^ DW the usual range reported in the bibliography. On the other hand, the Highland variety showed antioxidant activity of 11.9 mg TE g^−1^ DW with a usual range between 8.0 and 27.8 mg TE g^−1^ DW [61,69].

## 4. Conclusions

The present study consists of the optimization of a novel method for the UAE extraction and analysis of anthocyanins from purple potatoes. The Vitelotte variety was selected for this purpose. A Box-Behnken design (BBD) combined with response surface methodology (RSM) was employed for the optimization of four relevant variables, and the total yield of the four anthocyanins previously identified was used as the response variable.

The optimal conditions were established as 0.5 g of sample extracted using 20 mL of 60% methanol solvent in water at pH 2.9 at 70 °C with a cycle and amplitude of 0.5 and 70%, respectively. Finally, 15 min was established as the optimum extraction time. The repeatability and intermediate precision of the optimized methodology were confirmed by an RSD below 5% in all cases. In addition, re-extraction analysis was performed, but the results proved that one cycle of extraction with the optimized methodology is enough to ensure the maximum extraction of anthocyanins from purple potato samples.

After that, the developed method was applied to three purple varieties (Vitelotte, Double Fun and Violet Queen) and a red variety (Highland). The results corroborated the suitability of the optimized method for the detection and quantification of anthocyanins from purple and red potato samples. In addition, significant differences regarding anthocyanins content were observed.

Finally, the antioxidant activity of the extracts was measured using the DPPH method, and highly significant variety-related differences were observed. Furthermore, the developed method was confirmed to preserve the antioxidant activity of the anthocyanins extracted from the potato samples.

As previously mentioned, the composition of either raw material or elaborated products from purple potatoes may be altered or degraded as a result of certain processing methods or for other causes. For this reason, it would be really interesting to count on a rapid, reliable, economic and straightforward methodology to control the quality of the anthocyanins in these products.

## Figures and Tables

**Table 1:** Box-Behnken design experiment to optimize the extraction of total anthocyanins from purple potato.

Experiment	% MeOH	Temperature	pH	Ratio	Relative Area (Measured)	Relative Area (Predicted)	Relative Error in the Prediction (%)
1	0	−1	0	−1	72,953.22	74,783.20	2.45
2	1	0	0	−1	188,615.38	173,546.00	8.68
3	1	0	0	1	205,339.62	197,649.00	3.89
4	0	0	0	0	98,233.05	114,049.00	13.87
5	0	1	0	−1	209,930.07	209,546.00	0.18
6	0	0	−1	1	24,3104.45	263,197.00	7.63
7	0	0	1	−1	142,070.86	143,175.00	0.77
8	1	0	1	0	197,433.36	182,238.00	8.34
9	1	−1	0	0	120,781.49	127,148.00	5.01
10	0	1	−1	0	324,735.63	330,203.00	1.66
11	0	0	1	1	97,182.43	109,982.00	11.64
12	−1	0	−1	0	64,754.72	72,322.10	10.46
13	0	1	1	0	255,505.62	247,362.00	3.29
14	−1	0	0	−1	33,589.36	32,288.70	4.03
15	−1	1	0	0	68,379.59	63,210.00	8.18
16	0	0	−1	−1	187,493.78	195,891.00	4.29
17	0	−1	−1	0	236,810.85	201,385.00	17.59
18	1	1	0	0	348,856.93	316,545.00	10.21
19	0	−1	0	1	74,903.66	77,659.60	3.55
20	0	−1	1	0	77,330.88	78,294.80	1.23
21	−1	0	1	0	376.90	347.63	8.42
22	1	0	−1	0	282,293.09	316,194.00	10.72
23	−1	−1	0	0	41,213.04	45,278.10	8.98
24	0	0	0	0	116,928.90	114,049.00	2.53
25	0	0	0	0	96,983.88	114,049.00	14.96
26	0	1	0	1	260,240.38	240,782.00	8.08
27	−1	0	0	1	26,220.55	22,278.80	17.69

**Table 2:** Results from the BBD-RSM analysis on the anthocyanins obtained from purple potato samples.

Variable	Sum of Squares	*F* Value	*p*-Value
%MeOH	1.36 × 10^11^	41.92	0.00
Temperature	6.66 × 10^10^	20.52	0.00
pH	3.18 × 10^10^	9.81	0.01
Ratio	8.73 × 10^8^	0.27	0.61
%MeOH*%MeOH	6.57 × 10^9^	2.02	0.18
%MeOH*Temperature	1.64 × 10^9^	0.50	0.49
%MeOH*pH	9.60 × 10^8^	0.30	0.60
%MeOH*Ratio	4.97 × 10^7^	0.02	0.90
Temperature*Temperature	7.08 × 10^9^	2.18	0.17
Temperature*pH	4.05 × 10^8^	0.12	0.73
Temperature*Ratio	2.01 × 10^8^	0.06	0.81
pH*pH	2.17 × 10^10^	6.70	0.02
pH*Ratio	2.53 × 10^9^	0.78	0.39
Ratio*Ratio	2.07 × 10^5^	0.00	0.99
Error total	3.24 × 10^9^		

**Table 3:** Repeatability and intermediate precision results of the developed method.

	Repeatability	Intermediate Precision
Average	304,245.41	297,867.39
SD *	8012.67	10,154.10
RSD **	2.63	3.41

Repeatability (*n* = 8); intermediate precision (*n* = 24); * standard deviation; ** relative standard deviation.

**Table 4:** Concentration of anthocyanins (mg g^−1^ DW) and antioxidant activity (DPPH, mg TE g^−1^ DW) of purple and red flesh potatoes. Different letters on the same line mean that the values are significantly different.

Compound (mg/g DW)/Variety	Vitelotte	Double Fun	Violet Queen	Highland
1	n.d.	n.d.	n.d.	0.14 ± 0.00
2	0.66 ± 0.05 ^a^	0.59 ± 0.02 ^a^	3.82 ± 0.10 ^b^	n.d.
3	n.d.	n.d.	n.d.	1.12 ± 0.01
4	n.d.	n.d.	0.14 ± 0.01	n.d.
5	3.97 ± 0.14 ^c^	1.98 ± 0.09 ^b^	0.91 ± 0.09 ^a^	n.d.
6	n.d.	n.d.	n.d.	0.22 ± 0.00
7	0.34 ± 0.08 ^b^	0.21 ± 0.03 ^a^	n.d.	n.d.
Total anthocyanins (mg/g)	4.97 ± 0.17 ^c^	2.78 ± 0.10 ^b^	4.87 ± 0.13 ^c^	1.48 ± 0.01 ^a^
DPPH (mg TE/g DW)	28.81	21.67	29.87	11.19
Flesh Color	Purple	Purple/White	Purple	Red

1: Pelargonidin 3-*p*-coumaroylrutinoside-5-glucoside; 2: petunidin 3-*p*-coumaroylrutinoside-5-glucoside; 3: pelargonidin 3-*p*-coumaroylrutinoside-5-glucoside; 4: peonidin 3-*p*-coumaroylrutinoside-5-glucoside; 5: malvidin 3-*p*-coumaroylrutinoside-5-glucoside; 6: pelargonidin derivative; 7: malvidin 3-feruoylrutinoside-5-glucoside. n.d.: not detected.

## Data Availability

The data presented in this study are contained within the article and Supplementary Materials.

## Supplementary Materials

The following materials are available online at https://www.mdpi.com/article/10.3390/antiox10091375/s1. Figure S1: Chromatograms obtained for the potato variety Violet Queen by UHPLC-PDA-QToF-MS. The *m*/*z* identified were *m*/*z* 933.2390 (Time: 6.29 min: Compound 2. Petunidin 3-*p*-coumaroylrutinoside-5-glucoside), *m*/*z* 917.2419 (Time: 6.53 min: Compound 4. Peonidin 3-*p*-coumaroylrutinoside-5-glucoside), *m*/*z* 947.2563 (Time: 6.55 min: Compound 5. Malvidin 3-*p*-coumaroylrutinoside-5-glucoside), and *m*/*z* 977.2499 (Time 6.62 min: Compound 7. Malvidin 3-feruoylrutinoside-5-glucoside). Figure S2: Chromatogram obtained for the potato variety Highland by UHPLC-PDA-QToF-MS. The *m*/*z* identified was *m*/*z* 887.2387, corresponding with (Time: 5.92 min: Compound 1. Pelargonidin 3-*p*-coumaroylrutinoside-5-glucoside; Time: 6.46 min: Compound 3. Pelargonidin 3-*p*-coumaroylrutinoside-5-glucoside; Time: 6.59 min: Compound 6. Pelargonidin derivative). Figure S3: Overlay of the UHPLC chromatogram of the major anthocyanins in purple-fleshed Vitelotte (blue) and Violet Queen (red) potato varieties at 520 nm. **2**: Petunidin 3-*p*-coumaroylrutinoside-5-glucoside; **5:** Malvidin 3-*p*-coumaroylrutinoside-5-glucoside.
