# Supplementary material for: A Novel Ultrasound-Assisted Extraction Method for the Analysis of Anthocyanins in Potatoes (Solanum tuberosum L.)
Source: Antioxidants (Basel). 2021 Aug 28;10(9):1375. doi: 10.3390/antiox10091375 (PMC8468541; doi:10.3390/antiox10091375)
Supplement: Supplementary file 1 [file antioxidants-10-01375-s001.zip › antioxidants-1352501-supplementary.pdf]

antioxidants

MDPI

Article

A Novel Ultrasound-Assisted Extraction Method for the Analysis of Anthocyanins in Potatoes (*Solanum tuberosum* L.)

Supplementary Material

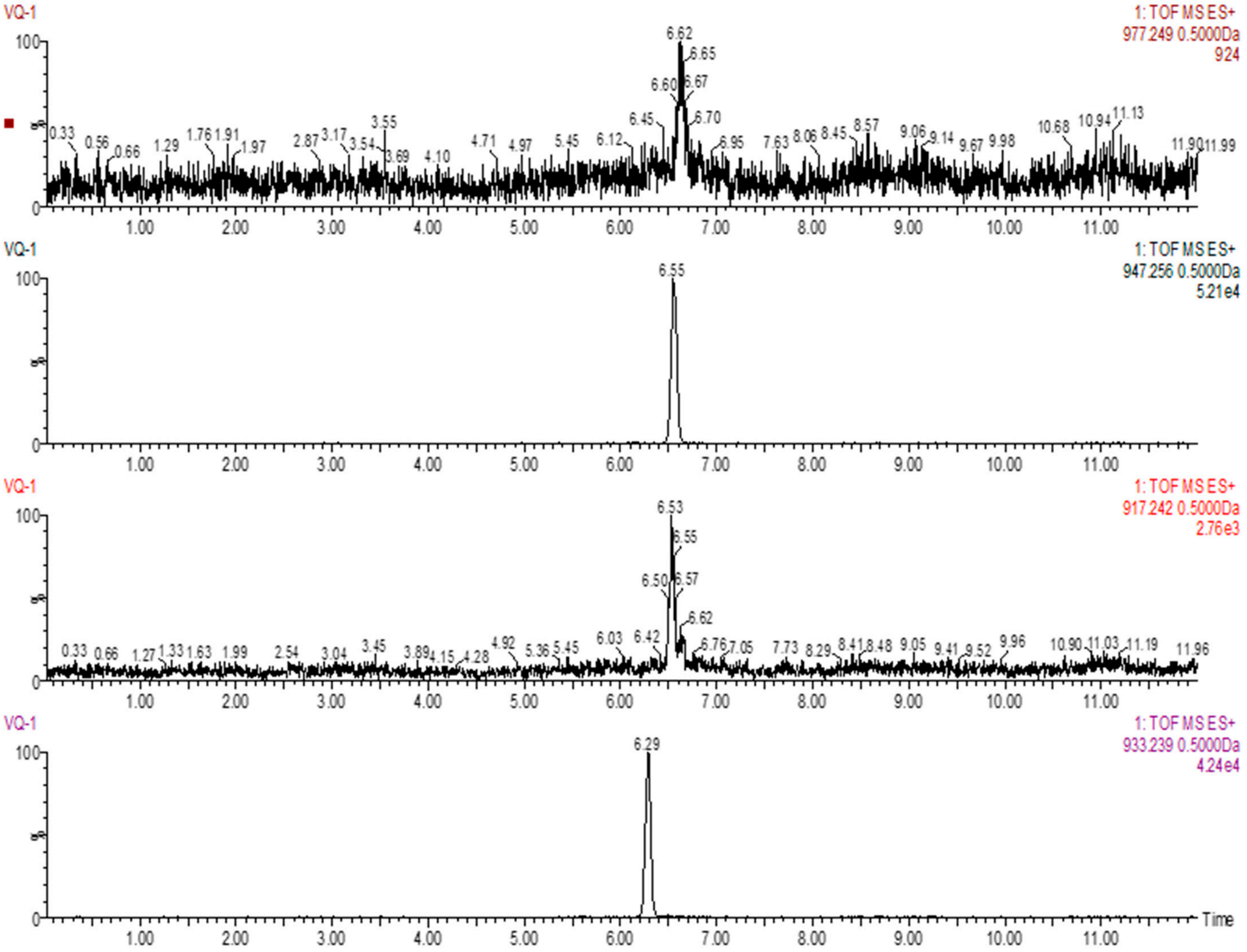

Figure S1. Chromatograms obtained for the potato variety Violet Queen by UHPLC-PDA-QToF-MS. The *m/z* identified were *m/z* 933.2390 (Time 6.29 min: Compound 2. Petunidin 3-*p*-coumaroylrutinoside-5-glucoside), *m/z* 917.2419 (Time 6.53 min: Compound 4. Peonidin 3-*p*-coumaroylrutinoside-5-glucoside), *m/z* 947.2563 (Time 6.55 min: Compound 5. Malvidin 3-*p*-coumaroylrutinoside-5-glucoside), and *m/z* 977.2499 (Time 6.62 min: Compound 7. Malvidin 3-feruoylrutinoside-5-glucoside).

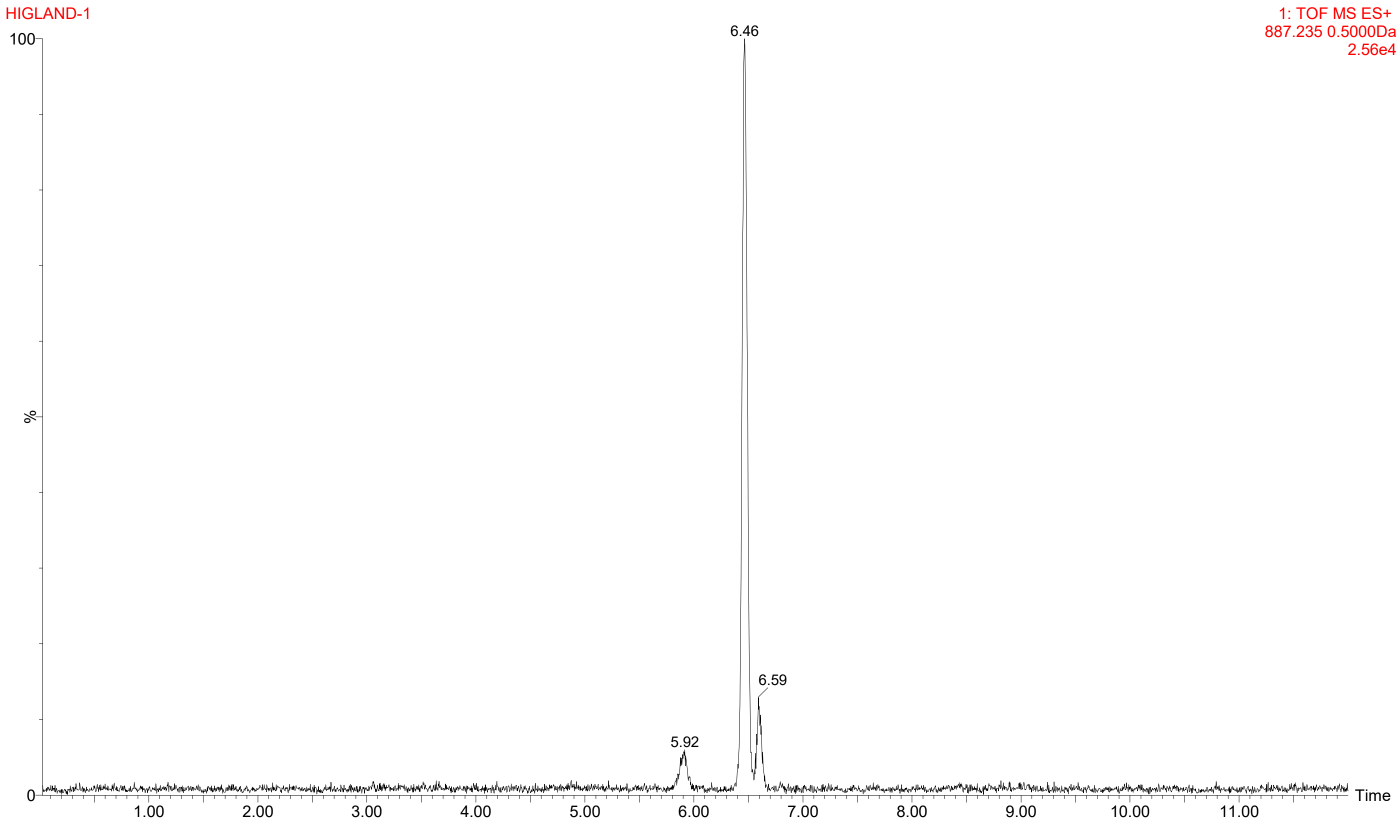

Figure S2. Chromatogram obtained for the potato variety Highland by UHPLC-PDA-QToF-MS. The *m*/*z* identified was *m*/*z* 887.2387, corresponding with (Time 5.92 min: Compound 1. Pelargonidin 3-*p*-coumaroylrutinoside-5-glucoside; Time 6.46 min: Compound 3. Pelargonidin 3-*p*-coumaroylrutinoside-5-glucoside; Time 6.59 min: Compound 6. Pelargonidin derivative).

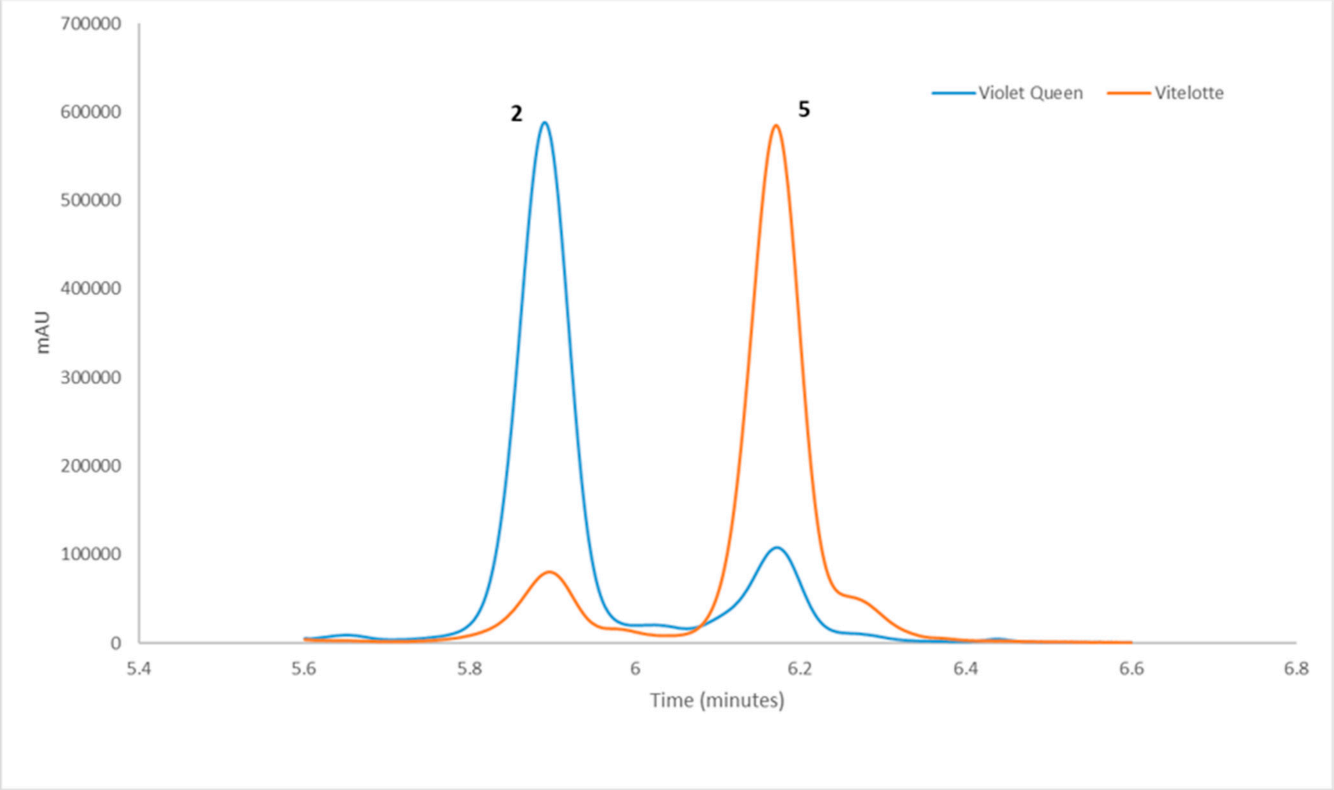

Figure S3. Overlay of the UHPLC chromatogram of the major anthocyanins in purple-fleshed Vitelotte (blue) and Violet Queen (red) potato varieties at 520 nm. **2**: Petunidin 3-*p*-coumaroylrutinoside-5-glucoside; **5:** Malvidin 3-*p*-coumaroylrutinoside-5-glucoside.
